# Supplementary material for: Natural Variation and the Role of Zn2Cys6 Transcription Factors SdrA, WarA and WarB in Sorbic Acid Resistance of Aspergillus niger
Source: Microorganisms. 2022 Jan 20;10(2):221. doi: 10.3390/microorganisms10020221 (PMC8877037; doi:10.3390/microorganisms10020221)
Supplement: Supplementary file 1 [file microorganisms-10-00221-s001.zip › microorganisms-1551021-supplementary.pdf]

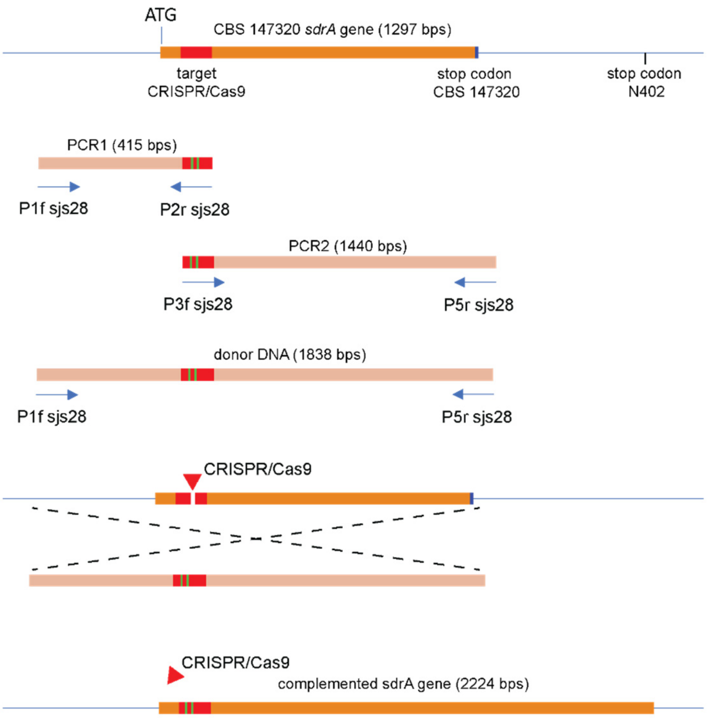

Figure S1. Complementation of the premature stop codon of *sdrA* in CBS 147320 using CRISPR/Cas9. A schematic overview of the complementation methodology with *sdrA* gene (orange), the location of the target used (red), the location of the stop codon in CBS 147320 (blue), the amplification method of the donor DNA (pink), with silent point mutations introduced (green) and the CRISPR/Cas9 complex (red triangle). PCR1 and PCR2 are performed using gDNA of N402 as a template, amplifying the version of *sdrA* without early stop codon using primer P5r sjs28. The primers P2r sjs28 and P3f sjs28 contain two silent point mutations to ensure that the CRISPR/Cas9 complex targeting the 23 bps on the original locus (red) does not recognize the target inside the donor DNA. During transformation, CRISPR/Cas9 creates a double-stranded break on the genome of CBS 147320 and homology-directed repair replaces the original locus with the provided donor DNA. In successful transformants CRISPR/Cas9 can no longer recognize and break open the complemented *sdrA* gene due to the two silent point mutations. As a result, the *sdrA* gene in CBS 147320 no longer contains an early stop codon.

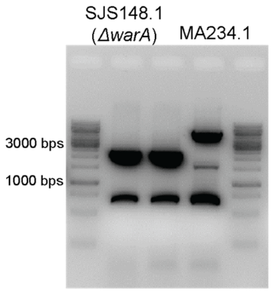

(a)

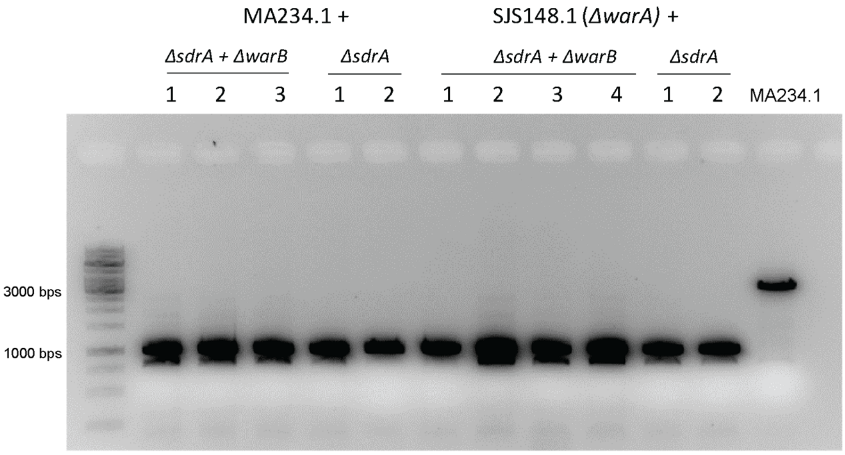

(b)

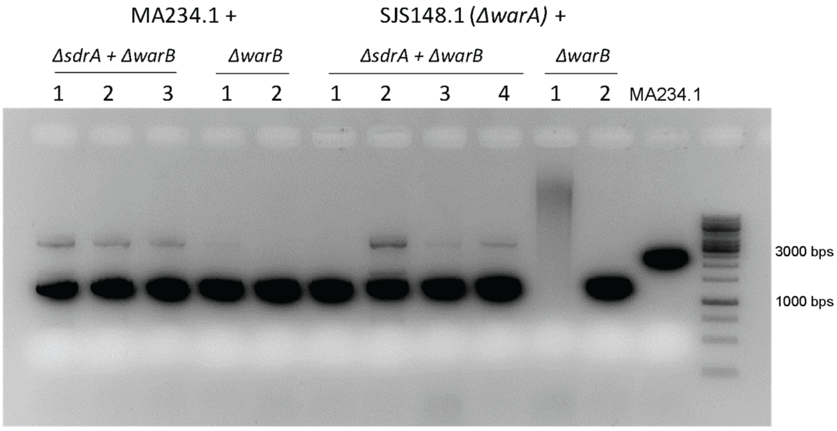

(c)

Figure S2. Diagnostic PCR confirming the *warA*, *sdrA* and *warB* deletions. (**a**) First, the *ΔwarA* deletion strain SJS148.1 was created. Diagnostic PCR was performed using forward primer DIAG_warA_fw and reverse primer DIAG_warA_rv to amplify the gene and flanking regions. A bandsize of 4800 bps is expected when *warA* is present, and a bandsize of 2166 bps is expected when *warA* is deleted. (**b**) Diagnostic PCR was performed using forward primer DIAG_sdrA_fw and reverse primer DIAG_sdrA_rv to amplify the gene and flanking regions. A bandsize of 3181 bps is expected when *sdrA* is present, and a bandsize of 964 bps is expected when *sdrA* is deleted. (**c**) Diagnostic PCR was performed using forward primer DIAG_warB_fw and reverse primer DIAG_warB_rv to amplify the gene and flanking regions. A bandsize of 2345 bps is expected when *warB* is present, and a bandsize of 1299 bps is expected when *warB* is deleted.

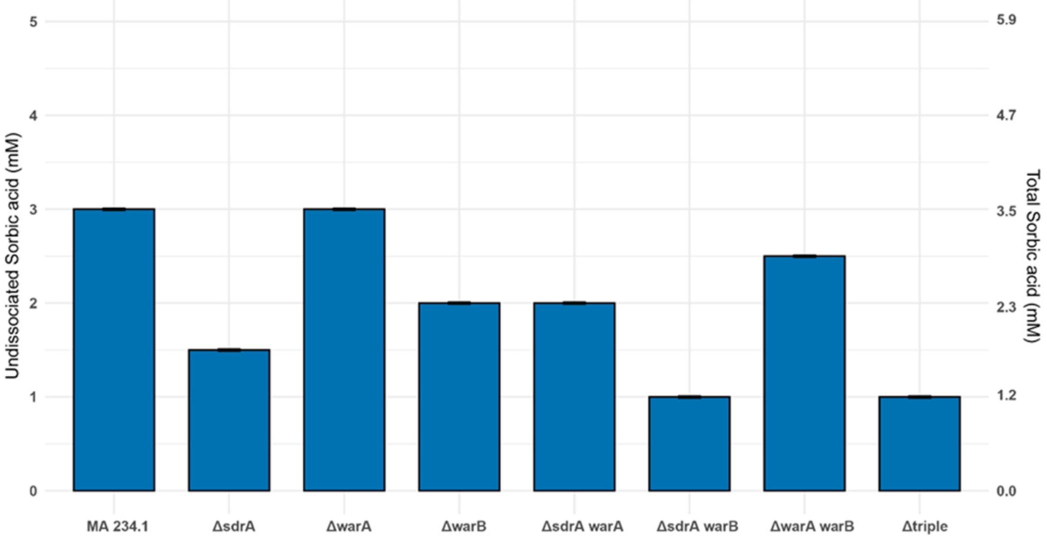

Figure S3. The sorbic acid MIC values in liquid MEB assay of the knock-out strains lacking *sdrA*, *warA* and/or *warB*. Average MIC values of transcription factor knock-out strains in liquid MEB (pH = 4). MIC of each strain was identified in biological duplicates. The mean MIC is visualized in blue, with the error bar indicating the standard deviation (all duplicates gave the same MIC so the standard deviations are 0 in all cases). The growth was scored after 4 days of growth at 30 °C. The primary Y-axis indicates the undissociated sorbic acid concentration, while the secondary Y-axis indicates the total sorbic acid concentration added.

Table S1. List of genes co-expressed with *warB*. Co-expression networks available on FungalDB [37ere used to determine which genes show significant co-expression with *warB*. A minimum Spearman coefficient of 0.5 was used to determine significant co-expression.

Gene ID	Spearman Coefficient	Product Description	Organism
An11g10870	1	Has domain(s) with predicted RNA polymerase II transcription factor activity, sequence-specific DNA binding, zinc ion binding activity, role in regulation of transcription, DNA-templated and nucleus localization	*Aspergillus niger* CBS 513.88
An15g06860	0.65	Ortholog of *Aspergillus nidulans* FGSC A4: AN7341, *Aspergillus oryzae* RIB40: AO090102000219, *Aspergillus fischeri* NRRL 181: NFIA_091700, *Aspergillus wenti*: Aspwe1_0107255 and *Aspergillus versicolor*: Aspve1_0125535	*Aspergillus niger* CBS 513.88
An02g14280	0.55	Ortholog of *S. cerevisiae*: TDA6, *A. nidulans* FGSC A4: AN7454, *A. fumigatus* Af293: Afu2g05980, *A. oryzae* RIB40: AO090001000716 and *A. wentii*: Aspwe1_0055229	*Aspergillus niger* CBS 513.88
An07g02180	0.55	E2 dihydrolipoamide acetyltransferase	*Aspergillus niger* CBS 513.88
An07g08890	0.55	Ortholog(s) have lipid-binding activity and role in eisosome assembly, endocytosis, negative regulation of protein kinase activity, protein localization, response to heat	*Aspergillus niger* CBS 513.88
An18g01150	0.55	Ortholog(s) have drug transmembrane transporter activity and role in cellular response to biotic stimulus, drug transmembrane	*Aspergillus niger* CBS 513.88

		transport, fluconazole transport, peptide transport, spermidine transport	
An01g05620	0.5	Has domain(s) with predicted hydrolase activity and role in metabolic process	*Aspergillus niger* CBS 513.88
An01g05630	0.5	Has domain(s) with predicted ATP binding, aminoacyl-tRNA ligase activity, aspartate-tRNA ligase activity, role in aspartyl-tRNA aminoacylation, tRNA aminoacylation for protein translation and cytoplasm localization	*Aspergillus niger* CBS 513.88
An02g04160	0.5	Mitochondrial phosphate translocator	*Aspergillus niger* CBS 513.88
An02g05210	0.5	Has domain(s) with predicted calcium ion binding, calcium-dependent phospholipid binding activity	*Aspergillus niger* CBS 513.88
An04g05360	0.5	Ortholog of *A. nidulans* FGSC A4: AN9473, *A. fumigatus* Af293: Afu4g11270, *A. oryzae* RIB40: AO090003001094, *A. wentii*: Aspwe1_0102365 and *Aspergillus sydowii*: Aspsy1_0148568	*Aspergillus niger* CBS 513.88
An07g04840	0.5	Has domain(s) with predicted hydrolase activity	*Aspergillus niger* CBS 513.88
An08g04300	0.5	Ortholog(s) have ubiquitin protein ligase binding activity, role in endocytosis, positive regulation of ubiquitin-dependent endocytosis, regulation of intracellular transport and early endosome, late endosome localization	*Aspergillus niger* CBS 513.88
An09g04300	0.5	Ortholog(s) have cytoskeletal protein binding, lipid binding activity	*Aspergillus niger* CBS 513.88
An11g09350	0.5	NADH-ubiquinone oxidoreductase	*Aspergillus niger* CBS 513.88
An12g09700	0.5	Has domain(s) with predicted oxidoreductase activity and role in metabolic process	*Aspergillus niger* CBS 513.88
An13g00200	0.5	protein of unknown function	*Aspergillus niger* CBS 513.88
An15g05450	0.5	Predicted mannitol dehydrogenase, expressed in conidiospores during sporulation	*Aspergillus niger* CBS 513.88
